# Editorial: Balloon and Stent for Ischemic and Hemorrhagic Stroke: A New Trend for Stroke Prevention and Management

**DOI:** 10.3389/fneur.2015.00218

**Published:** 2015-10-19

**Authors:** Firas Al-Ali

**Affiliations:** ^1^Department of Neuro Interventional Surgery, Akron General Medical Center, Akron, OH, USA

**Keywords:** acute ischemic stroke, brain anuerysm, intracranial angioplasty, intracranial stenting, atherosclerosis, capillary index score

It was the helium-filled balloons floating in the air during the 1959 International Workers’ Day in Moscow that first inspired Dr. Serbinenko to navigate from the common carotid artery to the intracranial circulation (1). He achieved that on February 8, 1964, by performing the first selective extracranial carotid injection with the assistance of temporary balloon for internal carotid artery occlusion. This was followed by the selective catheterization of the intracranial circulation using a flow-directed balloon catheter. Shortly after, he successfully treated a carotid-cavernous fistula (December 15, 1969) using a detachable balloon (1), marking the birth of our specialty: Neurointerventional Surgery.

Balloon catheters are no longer needed to achieve selective intracranial catheterization, but their therapeutic role has expanded significantly. Building upon the success of balloon angioplasty and balloon-mounted stents in the cardiology literature, Neurointerventionalists began using them intracranially, first for atherosclerotic stenosis (2), and then as an adjunct to coiling (3). Over the last 20 years, the applications and successes of these various balloons and stents have significantly expanded. We aim in this research topic to highlight the different trends being practiced today, some of which have the potential to become an integral part of our practice, while others may fade away.

The advent of aneurysm coiling (stand-alone coiling) has permanently changed the treatment of intracranial aneurysms, despite its limitations. The balloon remodeling technique (BRT) was first introduced in 1997 (3) and was followed by the stent-assisted coiling technique (SACT). Each has dramatically expanded the impact of aneurysm coiling, by allowing us to treat wide-neck aneurysms. Relying on their extensive experience, Dr. Piotin et al. detailed both BRT and SACT (4). They presented us with their mature technique and strategy concerning the use of each modality and device.

The different coiling techniques, however, suffer from two main shortcomings: aneurysm recurrence and an inability to treat giant aneurysms. Flow-diverters (FD) were recently invented to address these issues, for which Dr. Zanaty et al. gave a detailed introduction (5). Stemming from their title “Flow-Diversion Panacea or Poison?” it is apparent that there are still many questions about this technique, which they admirably try to address.

Another source of doubt and controversy in the care of subarachnoid hemorrhage patients is vasospasm prevention and treatment. Although it was first angiographically described in 1950 (6, 7), it is still a significant source of delayed morbidity and mortality. Dr. Bauer et al. detailed, in this research topic, the different methods of prevention and treatment as well as the controversy, but thankfully left us with clear and practical recommendations for day-to-day practice (8).

On another note, our knowledge of intracranial atherosclerotic disease (ICAD) is very limited, despite its prevalence, as Pu et al. highlighted in their review article. They emphasize, and rightly so, the stroke risk difference between symptomatic (approximately 10% per year) and asymptomatic ICAD (on the order of 3%) and the dynamic nature of the disease (9). Significant research on this subject is still needed.

Unfortunately, and despite its health burden worldwide, we still do not have a suitable treatment for ICAD. The EC-IC Bypass and SAMMPRIS trials failed to prove the benefits of surgical or endovascular treatments. We believe, however, that progress is still possible, especially in the pharmacology and endovascular realms. The authors of this research topic have already published some of the largest studies to date, relying on their extensive clinical experience. Concurrent with our distinguished panel, we believe that the reasons the endovascular treatment arm of the SAMMPRIS trial failed to show benefits are multifactorial, most of which can be corrected. First, the technique used was not meticulous enough as Connors et al. discuss in their paper here. They emphasize the importance of their previously described method (*Slow Inflation Undersized Balloon Technique*) in reducing technical complication rates (10). Second, only one device was allowed in the SAMMRIS trial (The Wingspan™, Stryker, Kalamazoo, MI, USA). McTaggart et al. argue in their paper in this research topic for intracranial “*angioplasty alone*” technique, mainly due to its safety profile in most cases (11), especially when coupled with Connors’ technique (10). Third, Miao presents, in his paper here, yet another critique to improve the SAMMPRIS trial results, where all lesions were treated identically (12). He successfully argues that each lesion is unique and should be treated differently, mainly based on lesion morphologies (*different lesion, different device*). For example, a concentric and short lesion could be treated by angioplasty alone, while other lesions need a more complex device. His *“Complex Strategy”* is very intriguing indeed.

Farooq et al. present a synthesis about the overall strategy of endovascular treatment regarding ICAD (13). They try to incorporate all authors’ recommendations, while placing an emphasis on the guiding catheter position *“the closer to the lesion, the better.”* They conclude with the assessment that a new trial, incorporating all these critiques and recommendations, is needed.

The final chapter of this research topic addresses acute ischemic stroke treatment. Al-Ali et al. argue that the presence or absence of collaterals (Circle of Willis and pial collaterals) determines the clinical outcome more than time from ictus to revascularization (14, 15): *“collaterals, not time, is brain.”* They argue for the use of the capillary index score (CIS) rather than an arbitrary time window to select patients for endovascular treatment. The CIS presumably reflects the percentage of viable tissue in the ischemic area, while its absence indicates non-viable tissue. The possibility that genetic factors play a determining role in the extent of collaterals, as emphasized by Dr. Faber (14), is a very exciting hypothesis and if proven will significantly impact the way we understand and treat ischemic strokes.

While in this research topic, we aim to present a synthesis of certain techniques practiced today; our true aim is to challenge our esteemed colleagues worldwide by raising more questions. We believe in their abilities and in the future of our promising specialty.

## Conflict of Interest Statement

The author declares that the research was conducted in the absence of any commercial or financial relationships that could be construed as a potential conflict of interest.
